# Evidence for enhanced multi-component behaviour in Tourette syndrome – an EEG study

**DOI:** 10.1038/s41598-017-08158-9

**Published:** 2017-08-10

**Authors:** Valerie C. Brandt, Ann-Kathrin Stock, Alexander Münchau, Christian Beste

**Affiliations:** 1 Department of Psychology, Centre for Innovation in Mental Health, University of Southampton, Southampton, UK; 2 Department of Paediatric and Adult Movement Disorders and Neuropsychiatry, Institute of Neurogenetics, Center for Brain, Behaviour and Metabolism, University of Lübeck, Lübeck, Germany; 3 Cognitive Neurophysiology, Department of Child and Adolescent Psychiatry, Faculty of Medicine of the TU Dresden, Dresden, Germany; 4 Experimental Neurobiology, National Institute of Mental Health, Klecany, Czech Republic

## Abstract

Evidence suggests that Tourette syndrome is characterized by an increase in dopamine transmission and structural as well as functional changes in fronto-striatal circuits that might lead to enhanced multi-component behaviour integration. Behavioural and neurophysiological data regarding multi-component behaviour was collected from 15 patients with Tourette syndrome (mean age = 30.40 ± 11.10) and 15 healthy controls (27.07 ± 5.44), using the stop-change task. In this task, participants are asked to sometimes withhold responses to a Go stimulus (stop cue) and change hands to respond to an alternative Go stimulus (change cue). Different onset asynchronies between stop and change cues were implemented (0 and 300 ms) in order to vary task difficulty. Tourette patients responded more accurately than healthy controls when there was no delay between stop and change stimulus, while there was no difference in the 300 ms delay condition. This performance advantage was reflected in a smaller P3 event related potential. Enhanced multi-component behaviour in Tourette syndrome is likely based on an enhanced ability to integrate information from multiple sources and translate it into an appropriate response sequence. This may be a consequence of chronic tic control in these patients, or a known fronto-striatal networks hyperconnectivity in Tourette syndrome.

## Introduction

Gilles de la Tourette syndrome (GTS) is characterized by multiple motor and vocal tics^1^ and associated with a number of structural and functional changes in fronto-striatal circuits^2–5^. Fronto-striatal circuits have been shown to be important for several cognitive functions. One of these is ‘multi-component behaviour’^6–12^; that is, the processing, prioritizing and integration of multiple actions, such as having a conversation while driving a car^13^. For this, information of different sensory modalities (e.g. visual and auditory information) has to be evaluated and integrated into actions^14^. Several lines of evidence suggest that fronto-striatal networks play an important role in action selection, or multi-component behaviour, and that basal ganglia dysfunctions, as well as dysfunctions in dopaminergic neural transmission impair these behaviours^7, 15–20^. On the basis of neurophysiological data (EEG), it has been demonstrated that this is due to processes mediating between stimulus evaluation and responding, i.e. response selection processes, which are reflected by the P3 event-related potential (ERP). Primary stimulus-related sensory and attentional processes associated with amplitude changes of P1 and N1 ERPs do not appear to play an important role in multi-component behaviour^21, 22^.

Interestingly, it has been argued that there is a relative fronto-striatal hyperconnectivity in GTS patients^2^. Moreover, GTS is associated with an imbalance or over-activity of the dopaminergic system^23^. Given increased striatal dopamine transmission^24–27^ and fronto-striatal hyper-connectivity in GTS^2, 4^, a counterintuitive hypothesis is that in this movement disorder multi-component behaviour is superior compared to healthy controls. If so, GTS patients are expected to show enhanced response selection processes (reflected by the P3 ERP), compared to controls. To examine multi-component behaviour and above-mentioned neurophysiological sub-processes, we used the ‘stop-change task’ (SCT) in GTS patients^6^. This task requires participants to occasionally interrupt (stop) an ongoing response in favor of an alternative response (change). Importantly, the signal to execute the alternative response is either presented at the same time, or with a short delay.

It is hypothesized that GTS patients will show enhanced response selection capacities during multi-component behaviour, especially when the change signal is presented simultaneously with the stop signal. This assumption is based on findings, showing that fronto-striatal mechanisms are particularly important under such circumstances^7, 12, 28^. We hypothesize that the expected group differences are not due to a simple speed-accuracy trade-off but should be based on Tourette specific neural alterations and should therefore be related to symptom severity.

## Results

### Clinical data

Out of 15 patients, 2 had an additional ADHD diagnosis and 3 had an additional OCD diagnosis. Questionnaire data and age is presented in Table 1. Six patients were taking tic-related medication (1 Paroxetin, 2 Abilify, 1 Medikinet, 1 Tegretal retard and 1 Abilify & Orap).

**Table 1:** Questionnaire data.

	YGTSS 50	YGTSS 100	PUTS	ADHD-SB	OCI-R	Age
GTS patients (n = 15) Mean ± SD	19.33 ± 9.15	34.33 ± 21.93	24.33 ± 12.11	9.27 ± 7.82	16.20 ± 15.49	30.4 ± 11.10
Healthy (n = 15) Mean ± SD	—	—	—	3.00 ± 2.93	6.80 ± 4.86	27.07 ± 5.44

Means and standard deviations (SD) of questionnaire data for patients and healthy controls. YGTSS = Yale Global Tic Severity Scale; PUTS = Premonitory Urge for Tics Scale; ADHD-SB = attention deficit hyperactivity disorder self-rating scale; OCI-R = obsessive-compulsive disorder scale revised.

### Behavioural results

**Figure 1:**
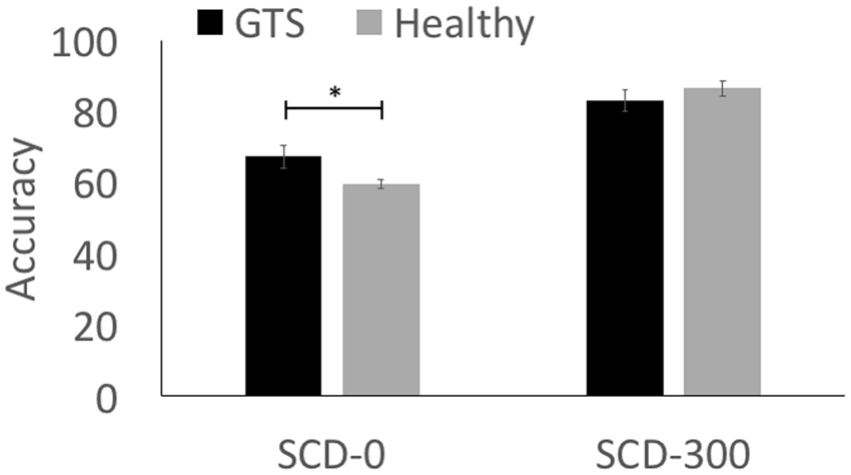
The figure displays accuracy (mean ± standard error) information for the stop change 0 ms delay condition (SCD-0) on the left and the 300 ms delay condition (SCD-300) on the right. *p < 0.05.

Regarding the STOP-CHANGE condition, a mixed effects ANOVA with the change conditions as the within-subjects factor [SCD-0, SCD-300] and group [controls, GTS patients] as the between-subjects factor showed that reactions in the SCD-300 condition (84.78 ± 1.89) were more accurate than reactions in the SCD-0 condition (63.45 ± 1.67) (*F*(1,28) = 144.43, *p* < 0.001, *ƞ*_*p*_^2^ = 0.84). There was a significant interaction ‘SCD x group’ (*F*(1,28) = 9.69, *p* = 0.004, *ƞ*_*p*_^2^ = 0.26), but no main effect ‘group’ (*F*(1,28) = 0.50, *p* = 0.48, *ƞ*_*p*_^2^ = 0.02). Post-hoc *t*-tests revealed that GTS patients (67.31 ± 3.12) reacted more accurately in the SCD-0 condition than the healthy control group (59.59 ± 1.22) (*t*(28) = 2.31, *p* = 0.03), while GTS patients (83.12 ± 3.13) did not differ from healthy controls (86.45 ± 2.13) in the SCD-300 condition (*t*(28) = −0.88, *p* = 0.39) (refer Fig. 1).

A post-hoc power analysis revealed that the achieved power in this interaction was higher than 99%.

For the reaction times (RTs) the mixed effects ANOVA showed that reactions associated with the SCD-300 condition were faster (918 ms ± 58.00) than reactions associated with the SCD-0 condition (1086 ± 56.61) (*F*(1,28) = 325.00, *p* < 0.001, *ƞ*_*p*_^2^ = 0.93). There was no significant interaction ‘SCD x group’ (*F*(1,28) = 0.04, *p* = 0.85, *ƞ*_*p*_^2^ = 0.001), but the main effect ‘group’ showed that GTS patients responded significantly slower (1139 ± 80.79) than healthy controls (866 ± 80.79; *F*(1,28) = 5.69, *p* = 0.02, *ƞ*_*p*_^2^ = 0.17). For the Go-condition, independent samples *t*-tests showed that healthy controls responded more accurately (*t*(28) = −3.32, *p* = 0.003), but not faster (*t*(28) = 1.89, *p* = 0.07) than GTS patients.

### Correlations regarding RTs and accuracy in the CHANGE conditions

RTs and accuracy did not correlate in GTS patients in the SCD-0 condition (*r* = 0.22, *p* = 0.42) or in the SCD-300 condition (*r* = 0.13, *p* = 0.65). Tic severity (YGTSS50) did not correlate significantly with accuracy in either condition (all *r* < 0.2) but did correlate significantly with RTs in the SC-0 condition (*r* = 0.65, *p* = 0.009) and the SC-300 condition (*r* = 0.65, *p* = 0.009).

### Neurophysiological results

#### P3 following STOP stimuli

**Figure 2:**
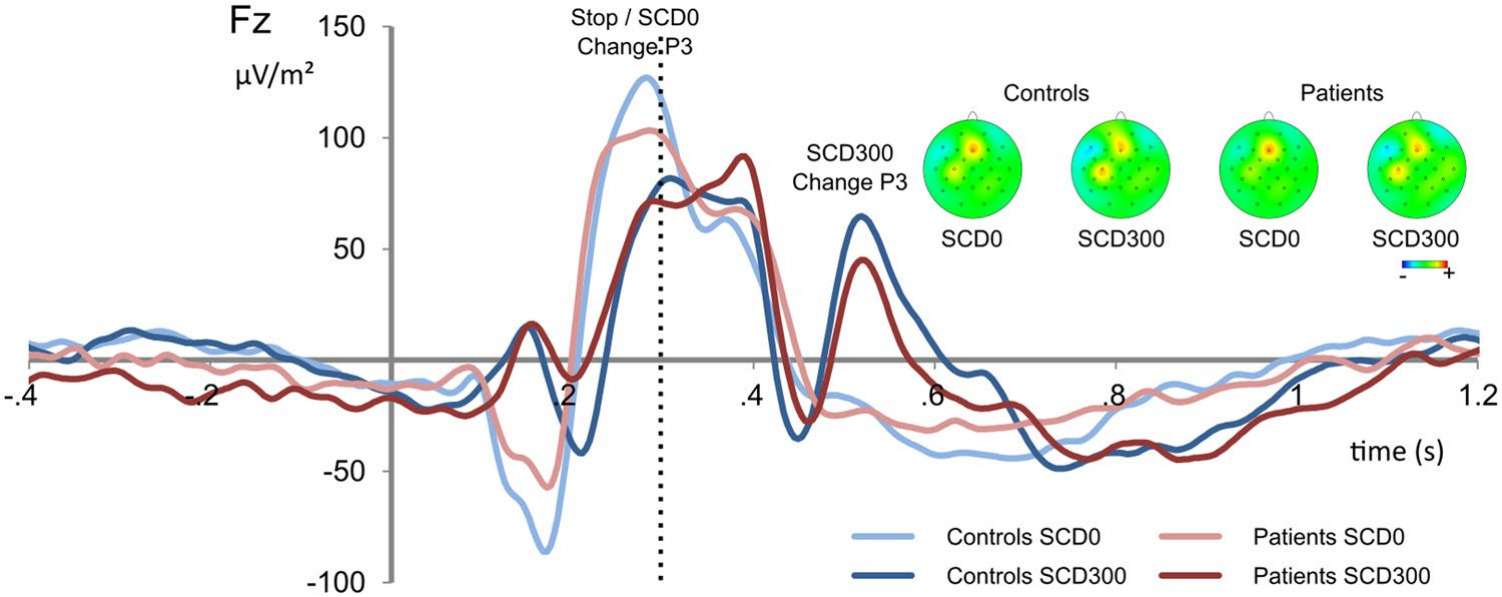
Event-related potential (ERP) components showing the P3 at electrode Fz. Time point zero denotes the time point of STOP stimulus delivery. Blue colors denote the control group, red colors the Tourette patient group. Lighter colors show the stop-change delay (SCD)-0 conditions, darker colors the SCD-300 condition. The scalp topography plots show the maximum amplitude of the P3 for controls and Tourette patients. Red colors show positivity, blue colors show negativity. The dashed vertical lines denote the time point of CHANGE stimulus delivery in the SCD-300 condition.

A mixed effects ANOVA [peak-to-peak P3 amplitudes at electrode Fz] with the change conditions as the within-subjects factor [SCD-0, SCD-300] and group [controls, GTS patients] as the between-subjects factor showed that the P3 amplitude was larger in the SCD-0 (183.72 ± 15.88) than in the SCD-300 condition (88.74 ± 10.8; F(1,28) = 42.66; *p* < 0.001; *ƞ*_*p*_^2^ = 0.604). Importantly, there was an interaction ‘SCD x group’ (F(1,28) = 5.86; *p* = 0.022; *ƞ*_*p*_^2^ = 0.173). Post-hoc *t*-tests showed that the P3 was larger in controls (211.26 ± 24.27) than GTS patients (159.67 ± 17.75) in the SCD-0 condition (*t*(28) = 1.74; *p* = 0.046) but not in the SCD-300 condition (*t*(28) = −0.89; *p* = 0.379). A post-hoc power analysis revealed that the achieved power in this interaction was 99% (see Fig. 2). It needs to be noted that there are differences in the distribution of sexes between the groups (i.e. 10 M/5 F in GTS patients and 3 M/12 F in healthy volunteers). Importantly, the pattern of results was unchanged when the factor “sex” was added to the model and there were no main or interaction effects of “sex” (all *F* < 2 *p* > 0.2).

#### P1 and N1 following visual STOP stimuli

**Figure 3:**
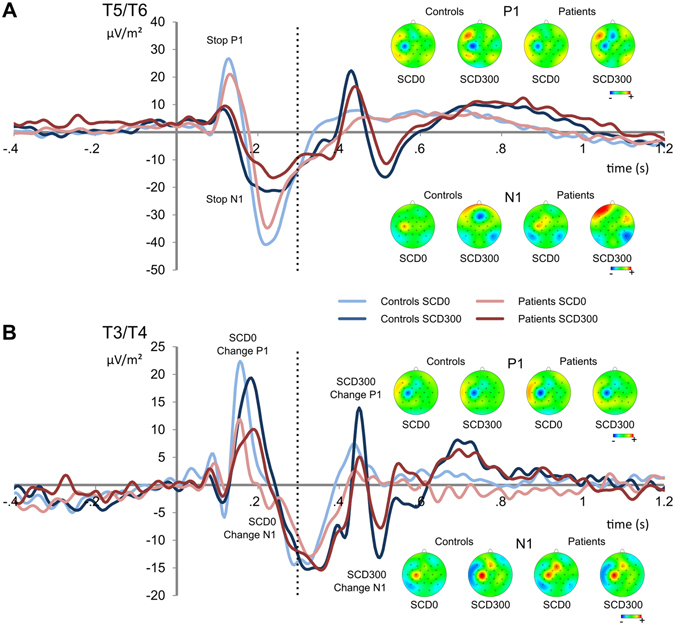
Event-related potential (ERP) components showing the P1 and N1 following the visual STOP stimuli (part A) and following the auditory CHANGE stimuli (part B). Time point zero denotes the time point of STOP stimulus delivery. Blue colors denote the control group, red colors the Tourette patient group. Lighter colors show the stop-change delay (SCD)-0 conditions, darker colors the SCD-300 condition. The scalp topography plots show the maximum amplitude of the P1 and N1 for controls and Tourette patients. Red colors show positivity, blue colors show negativity. The dashed vertical lines denote the time point of CHANGE stimulus delivery in the SCD-300 condition.

Mixed effects ANOVAs [P1 and N1] following visual STOP stimuli revealed that the P1 was larger for the SCD-0 (22.30 ± 2.83) than for the SCD-300 condition (9.0 ± 1.81; *F*(1,28) = 34.89; *p* < 0.001; *ƞ*_*p*_^2^ = 0.555). The main effect group (*F*(1,28) = 5.65; *p* < 0.025; *ƞ*_*p*_^2^ = 0.168) indicated that the P1 was larger in controls (20.64 ± 2.98) than GTS patients (10.66 ± 2.96). No other main or interaction effects with the factor ‘group’ were significant (all *F* < 2.03; *p* > 0.165). The N1 was largest (i.e. more negative) in the SCD-0 (−20.21 ± 2.34) than in the SCD-300 condition (−0.42 ± 1.17; *F*(1,28) = 71.87; *p* < 0.001; *ƞ*_*p*_^2^ = 0.720). The main effect ‘group’ (*F*(1,28) = 4.30; *p* = 0.047; *ƞ*_*p*_^2^ = 0.133) revealed that the N1 was larger in controls (−13.30 ± 2.03) than Tourette patients (−7.32 ± 2.03). The interaction ‘SCD x group’ was significant (*F*(1,28) = 5.04; *p* = 0.031; *ƞ*_*p*_^2^ = 0.153), but post-hoc tests did not withstand Bonferroni-correction (see Fig. 3A). The pattern of results was unchanged when the factor “sex” was added to the model and there were no main or interaction effects of “sex” (all *F* < 1.9 *p* > 0.2).

#### P1 and N1 following auditory CHANGE stimuli

Mixed effects ANOVAs [P1 and N1] following auditory CHANGE stimuli revealed only one significant main effect for the P1 [‘electrodes’], showing that the P1 was larger at electrode T5 (14.24 ± 2.53) than at T6 (8.15 ± 1.40; *F*(1,28) = 7.67; *p* = 0.010; *ƞ*_*p*_^2^ = 0.215). The N1 was also larger at electrode T5 (−30.64 ± 2.87) than at T6 (−15.89 ± 2.62; *F*(1,28) = 93.44; *p* < 0.001; *ƞ*_*p*_^2^ = 0.769). Moreover, the main effect ‘SCD interval’ (*F*(1,28) = 8.68; *p* = 0.006; *ƞ*_*p*_^2^ = 0.237) showed that the N1 was larger in the SCD-300 (−27.08 ± 2.84) condition than in the SCD-0 condition (−19.45 ± 3.04). No other main or interaction effects were significant and the pattern of results was unchanged when the factor “sex” was added to the model. There were no main or interaction effects with the factor “sex” (all *F* < 2.39; *p* > 0.133) (see Fig. 3B).

Together, the neurophysiological data showed an interaction effect between ‘SCD condition’ and ‘group’, specific to the P3 ERP-component. No interaction effects were evident for the P1 and N1 following the visual STOP or the auditory CHANGE stimuli.

## Discussion

In keeping with our main hypothesis, GTS patients reacted more accurately than healthy controls in trials requiring an immediate response change following the inhibition of a previously prepared response (SCD-0 condition). This was not explained by a speed-accuracy trade-off. As a neurophysiological correlate of superior behavioural accuracy in GTS the P3 ERP was smaller in immediate change trials in these patients. Smaller P3 amplitudes have previously been associated with better performance in the SCD-0 condition^7–12^. The results imply that GTS patients integrate multi-sensory (visual and auditory) information more efficiently during response selection processes^8, 29^, and therefore show better multi-component behaviour than healthy controls. Similar effects were not present regarding the P1 and N1 components, suggesting that purely sensory and attentional processes are unlikely to be responsible for the behavioural advantage shown by GTS patients in the SCD-0 condition.

The observed response selection advantage during multi-component behaviour in GTS may relate to a relative fronto-striatal hyperconnectivity in these patients^2, 4^. Especially the striatum and the thalamus appear to have abnormally strong structural connections with several frontal areas, including motor and sensory cortices, the supplementary motor area and the orbitofrontal cortex/frontal pole^3^. The neurophysiological data suggests that response selection processes (reflected by the P3 ERP) rather than sensory or attentional selection processes underlie the effects observed. Based on the importance of fronto-striatal loops for response selection processes^30–32^, it is possible that a fronto-striatal hyperconnectivity in GTS enhances response selection mechanisms. In this regard, it has been shown that performance in the SCD-0 condition depends more heavily on fronto-striatal mechanisms than in the SCD-300 condition^7, 12, 28^. Importantly, it has been shown that the P3, likely reflecting these response selection processes in this task, is modulated by striatal processes^7^. Performance in the task applied has been shown to be highly predictable on the basis of striatal GABA and glutamate concentrations^12^, which is in line with functional neuroimaging studies showing an involvement of striatal structures in this task^28, 33^. Moreover, modulation of the P3 in response selection tasks has been shown to be related to processes at the striatal level and, more generally, in fronto-striatal networks^34^. This may be one of the reasons why GTS patients’ multi-component behaviour advantage was particularly pronounced in the SCD-0 condition and reflected in the modulation of the P3. In contrast to the P3, the P1 and N1 ERP-components are well-known to be dependent on secondary visual and auditory cortices^35^ and are therefore not mediated by structures primarily affected in GTS.

Interestingly, there are other examples of enhanced cognitive control in GTS, relying on similar brain networks. In an anti-saccade task GTS patients outperformed healthy controls when switching between pro- and anti-saccades was required^36, 37^. It has been suggested that frequent tic suppression might increase executive control^36, 37^. This is because tic suppression activates frontal and motor networks^38^, as well as other areas in the cortico-striatal-thalamo-cortical loop^39^ that are also required for ‘cognitive flexibility’. Because of the disruptive nature of tics, GTS patients may frequently attempt to suppress or control their tics in certain social contexts and learn to execute other, less striking movements or response chains^40^. Therefore, GTS patients are in a chronic ‘multitasking’ state; they have to inhibit tics in favour of other movements or have to anticipate tics and work around them (e.g. interrupting a writing process to tic, then continue writing). It is therefore likely that GTS patients’ ability to integrate information from multiple sources and translate it into appropriate response sequences is highly developed. Multi-component behaviour is easier to execute in the SCD-300 than in the SCD-0 condition^41^ both for healthy controls and GTS patients. Performance advantages in task accuracy due to acquired abilities in GTS patients might therefore only become apparent in difficult task settings, such as the SCD-0 condition.

Another possible not mutually exclusive explanation for the observed response selection advantage during multi-component behaviour in GTS may relate to altered dopaminergic transmission in GTS because putatively increased dopamine levels enhance performance in multi-component behaviour^9, 10^. Interestingly, it has been shown that especially dopamine D1 receptor neural transmission is involved in the fast execution of learned responses^42^ and better performance as well as modulation of the P3 ERP in the task applied here^9^, while neural transmission via D2 receptors relates to controlled states during action selection^43^. However, tics are assumed to be mainly related to altered dopamine D2 receptors^44–48^, because blocking these receptors typically decreases symptom severity in GTS patients^49^. On the other hand, abnormalities in the D1 system have also been implicated in the pathophysiology of GTS^50, 51^ and may play a role for the effects described here. In GTS patients, there may be a link between superior skills to flexibly shift between response options and the relevance of specific dopaminergic systems for multi-component behaviour. This link should be investigated more directly in future studies.

There was a correlation between tic severity and response time, indicating longer response time in more severely affected patients in both the SCD-0 and SCD-300 condition. Despite the group level advantage of the GTS group in accuracy, it is possible that more severely affected patients found the task to be more effortful due to tics.

A limitation of this study is the limited sample size and that no comparisons between medication profiles in GTS patients were possible. However, the sample size is not a strong limitation, since the post-hoc power analysis revealed a power greater than 99% for observed interactive effects.

In summary, response selection during multi-component behaviour is more efficient in GTS compared to healthy controls. This benefit is not explained by altered perceptual or attentional processes but might be caused by fronto-striatal hyper-connectivity in these patients. Whether this is a consequence of chronic tic control and higher multi-tasking demands in these patients, i.e. adaptive, or inherent to GTS per se needs to be established.

## Methods

### Sample

Overall, N = 15 GTS patients (mean age = 30.40 ± 11.10; 10 male) diagnosed according to DSM 5 criteria^1^ and N = 15 healthy controls (mean age = 27.07 ± 5.44; 3 male) participated in this study. All patients were recruited by a GTS specialist at the University Hospital Schleswig-Holstein, Campus Lübeck, Germany (UKSH Lübeck). All participants gave their written informed consent to participate in the study. The experimental protocol was approved by the ethics committee of the UKSH Lübeck and was conducted in accordance with the local ethical guidelines as well as the declaration of Helsinki.

### Clinical assessment

GTS symptom severity and subjective impairment were assessed using a clinician-rated structured clinical interview, the Yale Global Tic Severity Scale (YGTSS)^52^. Current severity of possible comorbid attention deficit hyperactivity disorder (ADHD) symptoms was assessed with the German ADHD self-rating scale (ADHD-SB)^53^. Possible comorbid symptoms of obsessive-compulsive disorder (OCD) were measured using the revised version of the obsessive-compulsive Inventory (OCI-R)^54^. Premonitory urges were assessed using the validated German version^55^ of the ‘Premonitory Urge for Tics Scale’ (PUTS)^56^.

### Task

**Figure 4:**
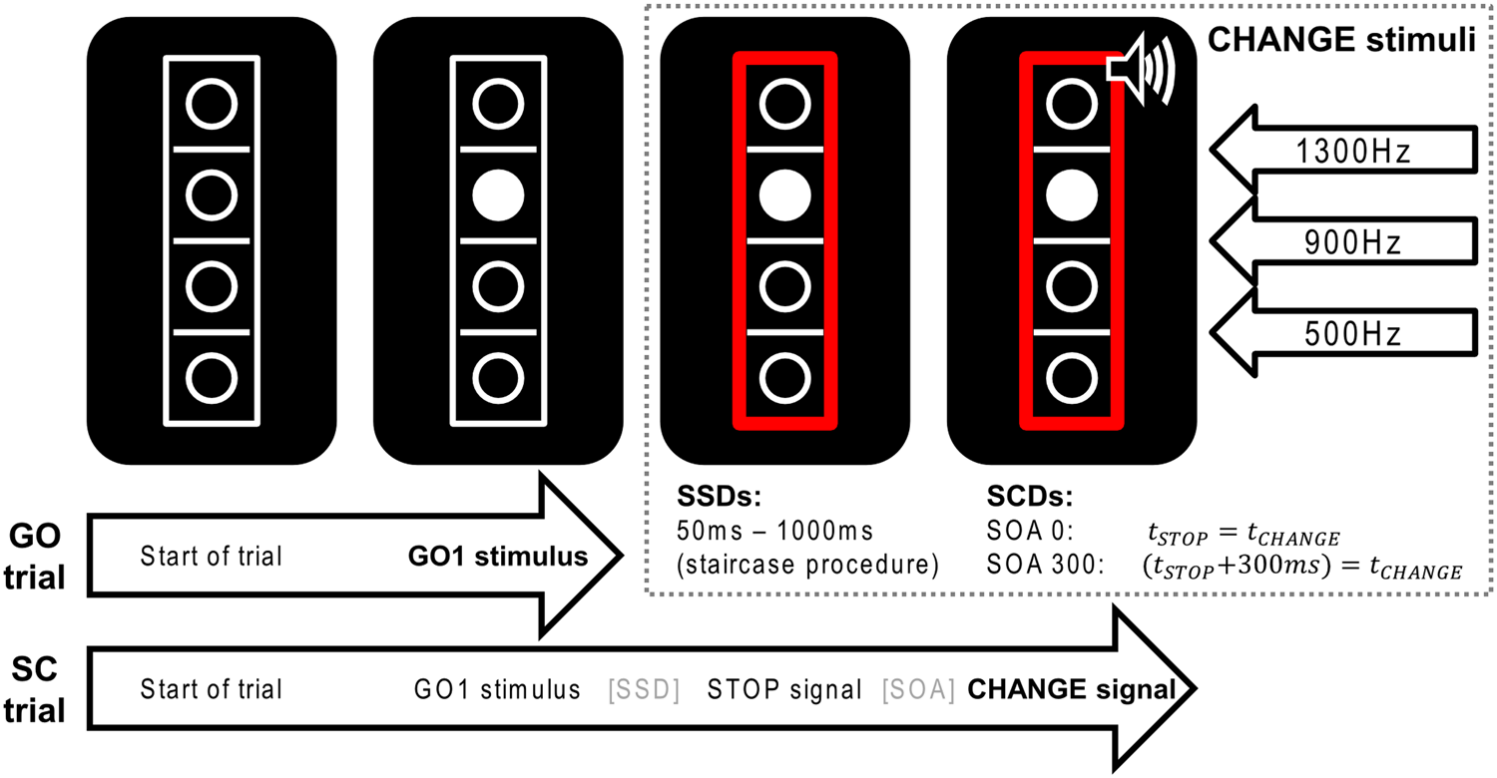
Stop-change paradigm: GO trials end after the first response to the GO stimulus, SC trials after a response to the CHANGE signal (bold). Delays between STOP (red rectangle) and CHANGE stimuli were 0 or 300 ms. Reference lines represent CHANGE stimuli.

This study employed a modified version of the SCT^6^ that has been used in several previous studies in adults^8^ and is shown in Fig. 4.

For extensive details of the task, the reader is kindly requested to refer to the above-mentioned published work. Briefly, the task was as follows: Every trial started with a white rectangle that was presented on a black background in the center of the screen. The rectangle was divided into 4 equally sized squares by horizontal reference lines. Each square contained the white outline of a circle. When one of the two inner circles switched to a solid white circle this became the target GO1 stimulus. Participants were asked to respond with their right hand to indicate whether the target was located above (right middle finger) or below (right index finger) the middle reference line. There were 576 GO trials. The remaining 288 trials were Stop-Change delay (SCD) trials. In these trials, the ongoing GO response was interrupted by a stop signal, represented by the white rectangle turning red. The stop signal was presented in different time intervals relative to the GO signal and it was ensured that 50% of GO trials could successfully be interrupted in each individual. Each STOP signal was accompanied by an auditory CHANGE signal (sine wave tone) that required a response with the left hand (GO2). This was either presented simultaneously with the STOP signal (SCD-0 condition) or 300 ms thereafter (SCD-300). Particularly in the SCD-0 condition, multi-component behaviour is difficult because STOP and CHANGE stimuli have to be processed simultaneously. The pitch of the tone was either high, medium, or low and represented one of the three reference lines within the rectangle. Participants were instructed to report whether the solid white circle was presented above or below the reference line ‘denoted’ by the respective CHANGE signal using their left hand. SCD-0 and SCD-300 were presented equally frequently. Trials of the different conditions were presented in a randomized order and participants were instructed to respond as fast and accurately as possible.

### EEG recording and analysis

The EEG was recorded using a Walter EEG system amplifier (Inomed Medizintechnik GmbH) with 32 Ag–AgCl electrodes at standard scalp positions. The reference electrodes were located at the right and left mastoid. The data were recorded with a sampling rate of 512.6 Hz and later (offline) down-sampled to 256 Hz. All electrode impedances were set to <5 kΩ. After recording, a band- pass filter ranging from 0.5 to 20 Hz was applied and manual inspection of the data was performed to remove technical artifacts. Next, the EEG data was segmented according to the two SCD conditions (SCD0 and SCD300). The segmentation was performed in relation to the occurrence of the STOP signal^8^. After the data was epoched, ocular artifacts were corrected using the Gratton-Coles algorithm^57^. These ocular-corrected epochs were then subjected to an artefact-correction procedure. The rejection criteria included a maximal value difference of 200 μV in a 250 ms interval, or activity <0.5 μV during an interval of 200 ms within the epoch. In order to eliminate the reference potential, a current source density (CSD) transformation was applied^58^ as previously done using 32 channel EEG data^59, 60^. The CSD also works as a spatial filter^61^, which helps to identify the electrodes that best reflect activity related to different cognitive processes. Then, a baseline correction was set to a time window of −900 to −700 ms to obtain a ‘real’ pre-stimulus baseline. Based on this stimulus locking procedure, the P1, N1, and P3 ERPs were quantified. Electrodes were chosen on the basis of visual inspection of the scalp topography, which showed a bilateral pattern of activation for the different ERP components. Due to this bilateral pattern, electrodes on both sides of the scalp were quantified. For this, the mean amplitude in a specific time interval was calculated. The visual P1 and N1 were measured at electrodes T6 and T7. This was done for the SCD0 and the SCD300 conditions between 120–130 ms for the P1 and between 210–220 ms for the N1. For the auditory P1 and N1 (measured at electrode T3 and T4) the quantification window was shifted according to the 300 ms SCD interval. Therefore, the time windows were as follows for the P1: 150–160 ms in the SCD0 condition and 450–460 ms in the SCD300 condition. Regarding the N1, the time windows were 190–200 for the SCD0 and 490–500 ms for the SCD300 condition. The P3 ERP-component was quantified at electrode Fz between 280–310 ms. Moreover, the negativity preceding the P3 was quantified to calculate peak-to-peak amplitudes.

### Statistical analysis

All variables were tested for normal distribution in each group (all Kolmogorov-Smirnov *Z* < 1.01, *p*s > 0.26). Mixed effects analyses of variance (ANOVAs) were used to analyse behavioural and ERP data, controlling for the factor “sex”. The STOP-CHANGE conditions (SCD0 and SCD300) were analysed separately from the GO condition. The factors ‘condition’ (SCD-0 trials and SCD-300 trials) and ‘electrode’ (only for ERP data) were used as within-subject factors. The factor ‘group’ (controls vs. GTS patients) was used as between-subjects factor. The degrees of freedom were adjusted using Greenhouse-Geisser correction. When necessary, all post-hoc tests were Bonferroni-corrected. For all descriptive statistics, means ± standard error are given. Correlations run between RT and accuracy data in the STOP-CHANGE conditions and symptom severity (YGTSS50) are Pearson’s *r*.

### Data availability statement

The datasets generated during and/or analysed during the current study are available from the corresponding author on reasonable request.
